# Aging and energetics’ ‘Top 40’ future research opportunities 2010-2013

**DOI:** 10.12688/f1000research.5212.1

**Published:** 2014-09-12

**Authors:** David B. Allison, Lisa H. Antoine, Scott W. Ballinger, Marcas M. Bamman, Peggy Biga, Victor M. Darley-Usmar, Gordon Fisher, Julia M. Gohlke, Ganesh V. Halade, John L. Hartman, Gary R. Hunter, Joseph L. Messina, Tim R. Nagy, Eric P. Plaisance, Mickie L. Powell, Kevin A. Roth, Michael W. Sandel, Tonia S. Schwartz, Daniel L. Smith, J. David Sweatt, Trygve O. Tollefsbol, Stephen A. Watts, Yongbin Yang, Jianhua Zhang, Steven N. Austad

**Affiliations:** 1 Office of Energetics, University of Alabama at Birmingham, Birmingham, USA; 2 School of Public Health, University of Alabama at Birmingham, Birmingham, USA; 3 Nutrition and Obesity Research Center, University of Alabama at Birmingham, Birmingham, USA; 4 School of Engineering, University of Alabama at Birmingham, Birmingham, USA; 5 Department of Environmental Health Sciences, University of Alabama at Birmingham, Birmingham, USA; 6 Department of Cell, Developmental, & Integrative Biology, University of Alabama at Birmingham, Birmingham, USA; 7 Department of Biology, University of Alabama at Birmingham, Birmingham, USA; 8 Department of Pathology, University of Alabama at Birmingham, Birmingham, USA; 9 Department of Medicine – Cardiovascular Disease, University of Alabama at Birmingham, Birmingham, USA; 10 Department of Genetics, University of Alabama at Birmingham, Birmingham, USA; 11 Department of Human Studies, University of Alabama at Birmingham, Birmingham, USA; 12 Department of Nutrition Sciences, University of Alabama at Birmingham, Birmingham, USA; 13 Comprehensive Center for Healthy Aging, University of Alabama at Birmingham, Birmingham, USA; 14 UAB Center for Exercise Medicine, University of Alabama at Birmingham, Birmingham, USA; 15 Department of Biostatistics, University of Alabama at Birmingham, Birmingham, USA; 16 Center for Free Radical Biology, University of Alabama at Birmingham, Birmingham, USA; 17 Birmingham VA Medical Center, Birmingham, USA

## Abstract

**Background**: As part of a coordinated effort to expand our research activity at the interface of Aging and Energetics a team of investigators at The University of Alabama at Birmingham systematically assayed and catalogued the top research priorities identified in leading publications in that domain, believing the result would be useful to the scientific community at large.

**Objective: **To identify research priorities and opportunities in the domain of aging and energetics as advocated in the 40 most cited papers related to aging and energetics in the last 4 years.

**Design: **The investigators conducted a search for papers on aging and energetics in Scopus, ranked the resulting papers by number of times they were cited, and selected the ten most-cited papers in each of the four years that include 2010 to 2013, inclusive.

**Results: **  Ten research categories were identified from the 40 papers.  These included: (1) Calorie restriction (CR) longevity response, (2) role of mTOR (mechanistic target of Rapamycin) and related factors in lifespan extension, (3) nutrient effects beyond energy (especially resveratrol, omega-3 fatty acids, and selected amino acids), 4) autophagy and increased longevity and health, (5) aging-associated predictors of chronic disease, (6) use and effects of mesenchymal stem cells (MSCs), (7) telomeres relative to aging and energetics, (8) accretion and effects of body fat, (9) the aging heart,  and (10) mitochondria, reactive oxygen species, and cellular energetics.

**Conclusion: **The field is rich with exciting opportunities to build upon our existing knowledge about the relations among aspects of aging and aspects of energetics and to better understand the mechanisms which connect them.

## Introduction

Energetics can be defined as the study of the causes, mechanisms, and consequences of the acquisition, storage, and utilization of metabolizable energy by biological organisms. The United States – indeed the world – is currently undergoing a crisis of excess energy storage, sometimes called the obesity epidemic. At the same time, the United States population is aging at an unprecedented rate with the over 65 population expected to double by 2050, the over 85 population expected to triple, and the over 100 population expected to more than sextuple (U.S. Administration on Aging; http://www.aoa.gov/Aging_Statistics/future_growth/future_growth.aspx#age). A consistent finding from ecology, basic laboratory science, and epidemiologic research is that aspects of energetics, including the perceived and actual availability of food, the ingestion of food, the composition of the food consumed, the amount of body energy accreted and expended, affect disease and disability, senescence, mortality rate, and longevity. A team of investigators at The University of Alabama at Birmingham (UAB) is currently aiming to advance innovative research at the interface of *aging and energetics* (hereafter A&E), and in preparation took stock of what ‘the field’ was describing as the vital research needs and opportunities in this domain. Realizing that the results of this assessment may be useful to others working in A&E, we publish them here.

## Methods/Approach

### Identification of the ‘Top 40’ Articles in A&E

To identify the ‘Top 40’ Articles in A&E, we conducted the following search in Scopus: ((TITLE((aging OR ageing OR lifespan OR longevity OR senescence)) AND PUBYEAR > 2009 AND PUBYEAR < 2014) AND (TITLE((calori* OR diet* OR energetic* OR nutri* OR food OR fat OR adipo* OR "body composition")) AND PUBYEAR > 2009 AND PUBYEAR < 2014)) AND (LIMIT-TO(LANGUAGE, "English")).

We then ranked the papers by number of times they were cited and selected the ten most cited papers in each of the four years that include 2010, 2011, 2012, and 2013. This ‘normalized’ for amount of time to be cited and resulted in a total of 40 papers ( Appendix A).

### Extraction of research priorities and themes

Ten of the authors then each reviewed 4 of the 40 top A&E publications and identified areas relative to A&E that were recommended by the original papers’ authors as needs for future research. For example, we extracted statements from the publications that included phrases like “More studies are needed on…”, “Future work should…”, “Further studies are needed to …”, “…not yet been established …”, and so on. Thereafter, two authors (DBA and LHA) grouped these statements into thematic categories. This involved some subjectivity and loss of some recommendations which did not easily fit in any category, but seemed to capture the vast majority of the recommendations. The ten thematic categories were labeled: 1)CR longevity response, 2) role of mTOR and related factors in lifespan extension, 3) nutrient effects beyond energy (especially resveratrol, omega-3 fatty acids, and selected amino acids, 4) autophagy and increased longevity and health, 5) aging-associated predictors of chronic disease, 6) use and effects of MSCs, 7) telomeres relative to aging and energetics, 8) accretion and effects of body fat, 9) the aging heart, and 10) mitochondria, reactive oxygen species, and cellular energetics. Specific suggestions often appeared in more than one category.

### Narrative description

We now *briefly* summarize the recommendations in each category.

## CR longevity response

### Robustness and heterogeneity of the CR longevity response

It was in rats, in 1917, that caloric restriction (CR) was first documented to extend life ^1^. In the decades following, researchers studying model organisms as diverse as yeast, *Drosophila*, rodents, and primates have accumulated experimental evidence demonstrating that CR extends lifespan, slows the aging process, and/or improves healthy aging biomarkers ^2– 4^. Concurrently, repeated studies within select model species (e.g., rats) further supported the robustness of this response ^4^. And thus, with this mounting evidence, the anti-aging response to a protocol of 20 to 40% CR was largely considered to be a robust response, evolutionarily conserved across animals.

Intrigued by this perceived adaptive response to an obvious physiological stress, researchers began traversing the animal lineage, beyond the traditional laboratory model organisms, to validate the robustness of this response. Instead they found extensive heterogeneity across species in their ability to extend median or maximum lifespan in response to CR ^4^. Similarly, testing additional genetic strains of lab animals uncovered extensive variation in the response to CR within inbred, outbred, and wild-derived strains of nematodes and mice (currently over 80 strains tested) ^4– 6^. For example, while CR has a relatively robust response in rats and *C. elegans*, by causing nearly all strains tested to increase median or maximum lifespan (~30% increase in rats); in mice this is true for only a subset of strains ^4^. Rather, the effect of CR across mouse strains (particularly the ILSXISS) ranged from decreasing or having no effect to increasing lifespan, with an average increase of only ~15% ^4^. Furthermore, this heterogeneity may be underplayed due to bias in reporting the negative effects of CR on lifespan ^4^. Thus, despite the anti-aging response to CR being evolutionarily conserved, there is now considerable evidence for heterogeneity in either the presence or absence of the lifespan extension response, and the degree of the effect on lifespan.

Despite this variation, researchers are actively searching for conserved molecular mechanisms underlying this response, typically using the short-lived model organisms. Many molecular mechanisms are implicated in this response, but few have been or are found to be consistent across species. A generalized theme is that reprogramming of metabolism in the face of CR is key to achieving lifespan extension ^2^.

The current challenge is to understand the sources of this heterogeneity and to determine if the underlying molecular mechanisms identified in the short-lived model organisms can translate to humans ^2^. The heterogeneity of the CR response across mice strains depicts a role for genetic variation ^5^. Some of the variation among strains is linked to the ability to regulate fat metabolism, thereby highlighting the link between body composition and aging ^5, 7^. The genetic variation also points to the possibility for natural selection to act on this response, which may explain the heterogeneity across populations and species of non-model organisms. The variable results in repeated studies within a genetic strain points to exceptional sensitivity of the response to other environmental variables. Indeed mammals under CR are often more susceptible to pathogens and other environmental stresses (e.g., cold stress) which may vary among laboratories and studies ^4^. Finally, heterogeneity among strains likely results from differences in gene by environment interactions, such that the optimal CR protocol (e.g., level of CR, dietary composition, developmental timing of implementation, and consistency of the regime) for producing anti-aging effects may be unique for each genetic background or population ^4, 7^.

One important question to consider is what one might call ‘forced inactivity’ and consequent low energy expenditure and any healthspan/lifespan consequences in caged laboratory animals. While there is substantial heterogeneity in volume of voluntary exercise across strains ^8^, the main point is that lab mice will voluntarily run some amount (and up to 11 km/day) if given a wheel, and may reap healthspan benefits from it. The field as a whole may create animals (particularly rodents) predisposed to disease and early death because of forced inactivity (see, e.g., 9– 11).

Overall, CR is the most discussed intervention for extension of life--span. While largely, but not perfectly, evolutionarily conserved across animals, there is extensive heterogeneity in this response within and across species. Understanding the heterogeneity in the response to CR at both the organismal and molecular level will be essential for predicting the plausible effects of elements of a CR regime or its pharmaceutical mimics to heterogeneous human populations.

### Mechanisms of the CR longevity response

Traditional thinking about CR, developed primarily with laboratory rodents, held that a significant restriction of dietary calories relative to *ad lib* feeding universally increased longevity and that energy intake alone, rather than the amount of any particular dietary nutrient, modulated lifespan ^12, 13^. Recent research on an extended range of model organisms makes both of those general conclusions no longer tenable. Not only does the same CR regime that extends life in some genotypes of mice, flies, and yeast have no effect on longevity in other genotypes ^14– 16^, it even shortens life in some genotypes indicating less than perfect evolutionary conservation ^17, 18^. Also, new evidence clearly indicates that restriction of some nutrients, in particular methionine or protein generally, is sufficient for significant life extension across a range of organisms ^19– 22^. Consequently, in some cases, the term “CR” as a descriptor of a longevity-enhancing, reduced nutrient regime should probably be replaced with the more generic and previously widely-used term “dietary restriction (DR)”. The life-extending impact of nutrient reduction, where it is observed, can be reasonably referred to as the “DR effect”.

What can these new studies, employing a broader array of species, genotypes, and nutrient regimes than previously, suggest about mechanisms of life extension by *dietary* restriction? First, the lack of a life-extending effect, or even a life-shortening effect, of DR in some genotypes can be informative. For instance, in a series of recombinant inbred mouse strains, the ability of a strain to live longer under 40% CR was significantly heritable and related to metabolic efficiency ^5^. Mice that lost the least weight and preserved the most body fat under reduced calories were most likely to live longer ^7^. These results together suggest that allelic variation in energetic efficiency genes may underlie the DR effect. Similarly, variation in replicative longevity in a series of 166 yeast single-gene deletion strains implicated vacuolar pH, superoxide dismutase activity, and mitochondrial proteome homeostasis (= proteostasis) as key determinants of the response to DR. Follow-up studies in *C. elegans*, which has been evolutionarily separated from yeast for ~1 billion years, also implicate mitochondrial proteostasis in the DR effect ^18^. Together these findings suggest a key role for energetic efficiency as a central process for understanding how nutrient intake and processing affect health and longevity. Second, despite the promise of a unified, conserved mechanism for the DR effect suggested above, other evidence indicates more mechanistic variety. For instance, the growth hormone (GH)/IGF-1 axis is strongly implicated in the DR effect in mice such that: (1) reduced GH and IGF-1 signaling have been genetically associated with longer life, (2) DR in mice reduces signaling through these pathways, and (3) DR fails to increase longevity substantially in GH receptor knockout mice ^23^. By contrast, in *C. elegans* insulin/IGF signaling is not required for the DR effect – at least under most DR regimes ^24^. However, DR is implemented by at least 12 methods in worms, differing in the culture medium, food source, and the age at which restriction is initiated. Surprisingly, the genetic pathways implicated in the DR effect vary depending upon method, suggesting considerable mechanistic complexity for such a seemingly straightforward environmental manipulation.

What does this avalanche of new information on DR across species suggest for future research directions according to our most-cited recent papers? First, and with most consensuses, determining the impact on health and longevity of the relative intake of different nutrients as well as the timing and number of calories consumed needs refinement and re-evaluation across a range of species and genotypes ^2– 4, 17, 25– 30^. However true, this suggestion lacks focus and more helpful perhaps is the linking by several papers of the DR effect either directly or indirectly to metabolic efficiency ^5, 18, 26^ and the maintenance of proteostasis ^20, 25, 31, 32^. With respect to this latter process, the most commonly adduced mediator of proteostasis was modulation of mTOR activity ^2, 25, 26, 32, 33^. Herein lies the most focused suggestion for future research to arise from our most-cited papers.

## Role of mTOR and related factors in lifespan extension

The mTOR kinase integrates signals from nutrients, energy status, growth factors, and a variety of stressors and affects, among other things, the rate of protein synthesis and degradation via autophagy by a host of downstream effectors ^34^. Thus TOR inhibition is a major player in the management of cellular energy and the maintenance of the proteome with aging. A number of our top-cited papers emphasize that the link between health, aging, nutrition, and energetics would be dramatically advanced by increased knowledge of how various nutrient and caloric manipulations affect and integrate both upstream mediators and downstream effectors of TOR activity, particularly on a tissue-specific basis.

Rapamycin, a macrolide with immunomodulatory properties, and the product of the bacterium *Streptomyces hygroscopius*, was found to inhibit an evolutionarily conserved protein belonging to the phosphatidyl inositol kinase-related serine/threonine kinase family, referred to as mTOR, the mechanistic target of rapamycin ^2, 25, 26, 35^. Modulating the mTOR signaling network affects mRNA translation, transcription, autophagy, ribosomal biogenesis, metabolism and cell survival, proliferation, cell size and growth, endoplasmic reticulum stress signaling, and other stress responses ^26^. In mammals, CR, or reduced dietary protein or essential amino acid intake, can extend longevity, improve metabolic fitness, and increase stress resistance, at least in part via inhibition of mTOR activity ^25^. Nutrients (glucose, amino acids, especially leucine, and fatty acids) directly activate the mTOR pathway and also increase insulin levels, which can additionally activate mTOR ^35^. mTOR acts as a major signaling hub, integrating multiple inputs and mediating the switch between growth and somatic maintenance to extend lifespan ^26^. Mammalian cells have a single mTOR kinase which exists in two structurally and functionally distinct multiprotein complexes: mTORC1 and mTORC2. mTORC1 is acutely inhibited by rapamycin, although chronic rapamycin administration inhibits mTORC2 as well ^36^. Amino acid deprivation is also a powerful inhibitor of mTORC1 activity even the presence of acute growth factor stimulation ^25, 26^. Strong inhibition of mTORC1 early in life drastically slows or even stops development, but administration of rapamycin or other inhibitors of mTORC1 late in life causes lifespan extension, boosts immune function and rejuvenates hematopoietic stem cells ^25, 26^.

**Figure 1.**
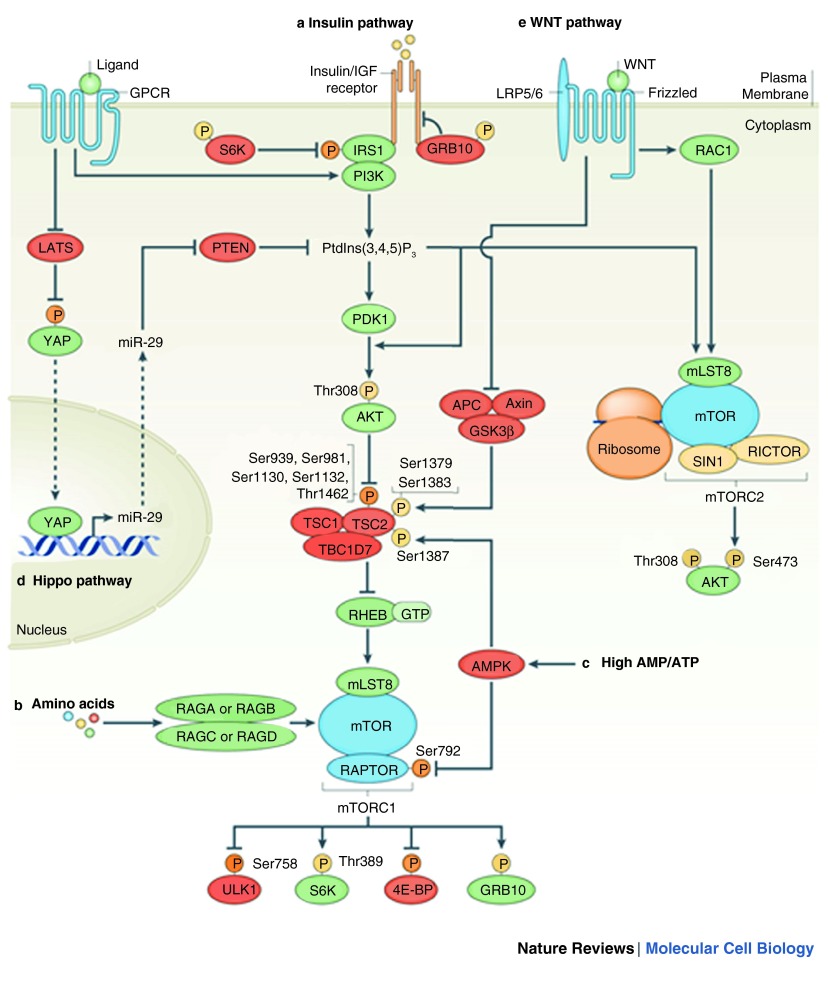
Upstream of mTOR: classical and non-classical inputs. **a** | Growth factors such as insulin stimulate PI3K to generate phosphatidylinositol-3,4,5-triphosphate (PtdIns(3,4,5)P _3_), which promotes the phosphorylation (P) of AKT at Thr308 by phosphoinositide-dependent kinase 1 (PDK1). AKT phosphorylates tuberous sclerosis complex 2 (TSC2) on multiple sites to inhibit its GTPase-activating protein (GAP) activity for the small GTPase RAS homologue enriched in brain (RHEB). GTP-loaded RHEB then activates mammalian TOR complex 1 (mTORC1). Growth factors also stimulate mTORC2 by promoting its association with ribosomes in a PI3K-dependent manner. **b** | Amino acids stimulate mTORC1 by promoting the conversion of RAS-related GTP-binding protein (RAG) heterodimers to the active conformation, in which RAGA or RAGB is loaded with GTP and RAGC or RAGD is loaded with GDP. **c** | In response to low energy (high AMP/ATP ratio), AMP-activated protein kinase (AMPK) phosphorylates regulatory-associated protein of mTOR (RAPTOR) at Ser792 and TSC2 at Ser1387, leading to the inhibition of mTORC1. **d** | During the inhibition of the Hippo pathway component large tumour suppressor homologue (LATS) kinase, hypophosphorylated Yes-associated protein (YAP) translocates to the nucleus and promotes the expression of the microRNA miR-29. miR-29 targets PTEN mRNA and inhibits PTEN translation, which leads to increased levels of PtdIns(3,4,5)P _3_ and the activation of both mTORC1 and mTORC2. Dashed arrows represent translocation of the molecule. **e** | Glycogen synthase kinase 3β (GSK3β) activates the TSC complex by phosphorylating TSC2 at Ser1379 and Ser1383. Phosphorylation of these two residues requires priming by AMPK-dependent phosphorylation of Ser1387. WNT signalling inhibits GSK3β and the TSC complex, and thus activates mTORC1. mTORC2 is activated by WNT in a manner dependent on the small GTPase RAC1. Proteins shown in green promote mTOR activity or their activity is promoted by mTOR. Proteins shown in red inhibit mTOR activity or their activity is inhibited by mTOR. Phosphorylation depicted in yellow is an activation signal and phosphorylation depicted in orange is an inhibitory signal. 4E-BP, eIF4E-binding protein; APC, adenomatous polyposis coli; GPCR, G protein-coupled receptor; GRB10, growth factor receptor-bound protein 10; IGF, insulin-like growth factor 1;IRS1, insulin receptor substrate 1; LRP, low-density lipoprotein receptor-related protein; mLST8, mammalian lethal with SEC thirteen 8; RICTOR, rapamycin-insensitive companion of mTOR; S6K, ribosomal S6 kinase; SIN1, SAPK-interacting 1; TBC1D7, TBC1 (TRE2–BUB2–CDC16) domain family member 7; ULK1, UNC-51-like kinase 1. This figure has been reproduced with kind permission from Shimobayashi M, Hall M. Making new contacts: the mTOR network in metabolism and signalling crosstalk. Nature Reviews Molecular Cell Biology 2014;15:155–162.

**Figure 2.**
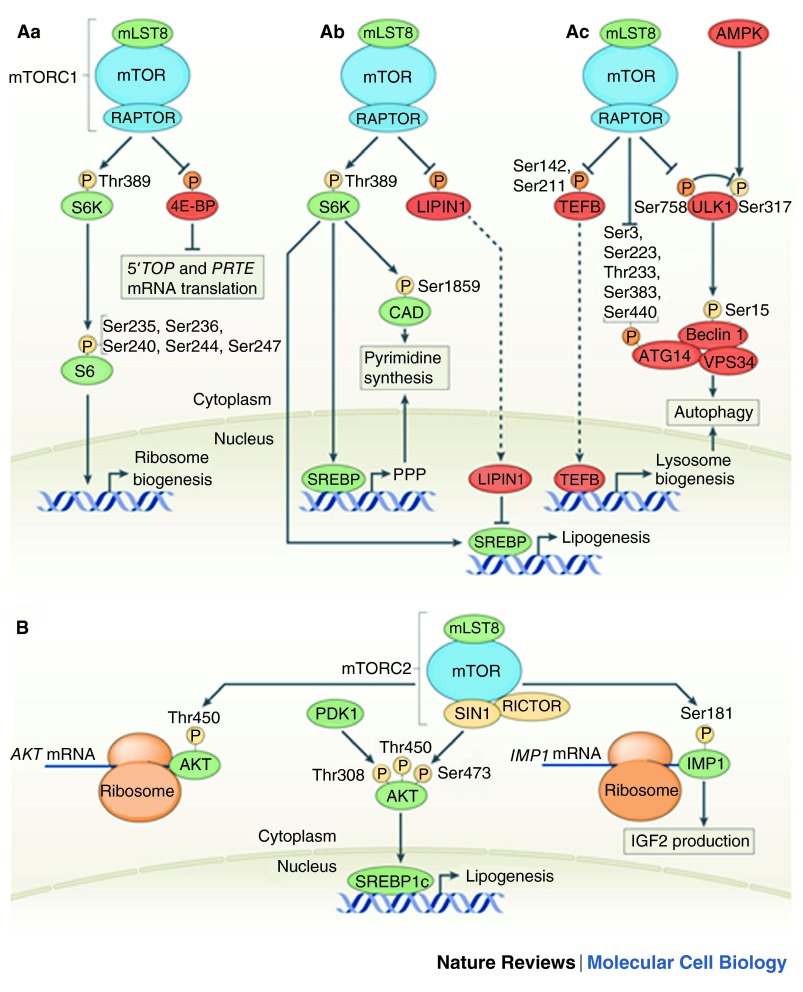
mTOR controls metabolism. Mammalian TOR complex 1 (mTORC1) promotes anabolic processes, such as the biosynthesis of proteins, nucleotides and lipids, and inhibits catabolic processes such as autophagy. **Aa** | mTORC1 phosphorylates (P) the hydrophobic motif (Thr389) in ribosomal S6 kinase (S6K), thereby activating it to subsequently phosphorylate ribosomal protein S6 at the sites indicated to promote ribosome biogenesis. mTORC1 also phosphorylates eIF4E-binding protein (4E-BP) at multiple sites to inhibit it. Inhibition of 4E-BP stimulates translation initiation, especially of 5′ oligopyrimidine tract (termed a 5′ TOP) and pyrimidine-rich translational element (PRTE) containing mRNAs. **Ab** | mTORC1 stimulates nucleotide and lipid synthesis. mTORC1 promotes the gene expression of key enzymes in the pentose phosphate pathway (PPP), at least in part by activating sterol regulatory element-binding proteins (SREBPs). mTORC1 also stimulates CAD (Gln-dependent carbamoyl-phosphate synthase, Asp carbamoyltransferase, dihydroorotase) by S6K-mediated phosphorylation at Ser1859, which leads to CAD activation and the stimulation of de novo pyrimidine synthesis. Furthermore, mTORC1 promotes lipogenic gene expression by activating S6K or by inhibiting the nuclear translocation of LIPIN1, both of which activate the transcription factor SREBP. **Ac** | mTORC1 inhibits autophagy by phosphorylating UNC-51-like kinase 1 (ULK1) at Ser758 and ATG14 at multiple sites. During mTORC1 inhibition, AMPK phosphorylates ULK1 at Ser317, and thereby activates ULK1, which phosphorylates Beclin 1 in the vacuolar protein sorting 34 (VPS34)–Beclin 1–ATG14 complex to initiate autophagy. mTORC1 also inhibits autophagy indirectly by blocking lysosome biogenesis, by phosphorylating and inhibiting the nuclear translocation of transcription factor EB (TFEB). **B** | mTORC2 co-translationally phosphorylates AKT at Thr450 to prevent its ubiquitylation and degradation. mTORC2 also post-translationally phosphorylates ATK at Ser473 to promote lipogenic gene expression by activation of SREBP1c. Moreover, mTORC2 co-translationally phosphorylates IGF2 mRNA-binding protein 1 (IMP1) at Ser181, which stimulates insulin-like growth factor 2 (IGF2) production. The activity of proteins shown in green is promoted by mTOR. The activity of proteins shown in red is inhibited by mTOR. Phosphorylation depicted in yellow is an activation signal and phosphorylation depicted in red is an inhibitory signal. Dashed arrows represent translocation of the protein. mLST8, mammalian lethal with SEC thirteen 8; PDK, phosphoinositide-dependent kinase 1; RAPTOR, regulatory-associated protein of mTOR; RICTOR, rapamycin-insensitive companion of mTOR; SIN1, SAPK-interacting 1. This figure has been reproduced with kind permission from Shimobayashi M, Hall M. Making new contacts: the mTOR network in metabolism and signalling crosstalk. Nature Reviews Molecular Cell Biology 2014;15:155–162.

The specific upstream mechanisms directly responsible for amino acid sensing which are permissive of mTOR activation remains unresolved, but localization of the mTORC1 complex has emerged as an important aspect of amino acid mediated control ^25^ (see Figure 1 and Figure 2). The mTORC1 pathway controls protein synthesis most directly by phosphorylating and inhibiting a repressor of cap-dependent mRNA translation, 4E-BP1. mTORC1 also regulates translation indirectly through activation of S6 kinase (S6K) whose substrate is the ribosomal protein S6, a component of the 40S ribosome important for translation ^26^.

A drop in the cell’s energy content is reflected in the rise of the AMP/ATP ratio activating AMPK, which reduces the activity of mTORC1 by direct phosphorylation of TSC2 (Tuberous Sclerosis Complex 2), a negative regulator of mTORC1, and Raptor, an essential binding partner for the induction of translation by mTORC1 ^2, 26^. Biochemical evidence places AMPK upstream of mTORC1, but AMPK has also been shown to be downstream of S6K and can mediate S6K dependent effects on lifespan. Similarly, S6K is directly downstream of mTORC1, but via its inhibitory effect on IRS1 and 2, it is also upstream of mTORC1 via an insulin-signaling route ^34^. The complex biochemical interactions among the various players in the mTOR network clearly require more refined mechanistic investigation ^26^. Thus, mTORC1, S6K and AMPK may constitute a complex feedback loop sensitive to dietary restriction which can redirect growth, metabolism and lifespan. In addition to nutrients and growth factors, appropriate control of growth also requires integration of information on environmental stresses. Osmotic stress, hypoxia, ER stress, genotoxic stress, mechanical forces, and contraction (e.g., muscle movement) may all regulate mTORC1 activity ^26^.

Key questions that still need to be delineated are how multiple nutrients, growth factors and stress inputs are integrated into a single mTORC1 activity level; how the physiological responses downstream of mTORC1 are differentially regulated; how mTOR mediates changes in survival and lifespan in response to various environmental manipulations, especially nutrients; how the sex-dependent effects of mTORC1 signaling on longevity are regulated; with multiple mechanisms proposed; how mTORC1 activity couples nutrient availability to ribosome biogenesis; and which transcription factors involved in mediating stress responses are regulated by mTORC1 signaling to affect lifespan extension? Furthermore, the downstream effectors of mTORC1 are not sufficiently determined to understand which pathways are absolutely necessary for mTORC1’s role in lifespan extension. The role of mTORC1 is likely to be highly tissue specific, and the communication between cells, tissues, and organs to determine lifespan is a major knowledge gap. Lastly, little is known about the role of mTORC1 in primate aging and life extension by CR in those primate populations in which such extension is seen ^2, 25, 26, 28, 35^.

## Nutrient effects beyond energy (especially resveratrol, omega-3 fatty acids, and selected amino acids)

In addition to the well-recognized effect of CR on longevity and disease, there is growing evidence that the abundance of individual nutrients, macronutrient types, and nutritional supplements (i.e., non-caloric dietary compounds) specifically influence health and longevity. For example, restriction of the essential amino acid, methionine, can improve health and increase longevity in mice and rats, although importantly too much restriction can compromise health as well ^21, 37, 38^. Methionine restriction provides benefits independent of caloric intake (i.e., with *ad lib* feeding). Although mechanistic differences are not fully understood, one possibility is that specific pathways sensitive to dietary nutrients can be targeted to alter lifespan. Examples include amino acid signaling with the mTOR pathway, energy status sensing through the adenosine monophosphate kinase (AMPK) pathway, oxidative stress through SKN1/Nrf2, protein quality control though enhanced autophagy and the general stress response. A variety of perturbations may activate ‘longevity’ pathways such as these to promote better health.

There is also evidence accumulating that plant-based, bioactive compounds (phytochemicals) have a variety of cellular effects and also work through multiple pathways. While a focused approach is helpful for establishing cause-effect for individual compounds, it is becoming clear that we must understand combinations of dietary effects and their interactions to fully describe the biological complexity underlying their contributions to homeostatic control and cellular regulation. For instance, one of the most well-known, and highly-researched CR mimetics (compounds which are proposed to mimic the benefits of CR without the necessity of energy intake reduction) is resveratrol, a polyphenol found in the skins of red grapes, muscadines, etc. Resveratrol is proposed to possess multiple agonist (sirtuins, AMPK) and antagonist (TOR/S6K, heat shock response) activities to pathways which have been genetically demonstrated to mediate longevity and health outcomes. Yet, the effects of resveratrol remain open to question. Resveratrol has not yielded consistent results on longevity outcomes, but it remains a highly researched phytochemical that may mediate disease risk or cellular/tissue health through other of these multiple interactions. Mice appear “largely unaffected by resveratrol” ^40^.

An alternative approach to pathway specific targeting is exemplified in omega-3 fatty acid research. Polyunsaturated fatty acids (PUFAs), which contain double bonds in their carbon backbone, can be subdivided in various families, with the ‘omega-3’ (double bond at the n-3 position) type PUFA garnering the most health claims. This may be related to the change in the structure and biochemical properties of the lipid that provides important components of cellular membranes and protection from oxidative stress, particularly in the central nervous system where omega-3s are concentrated in nervous tissues. Although dietary deficiency has been associated with neurodegenerative disease risk and accelerated aging, supplementation with omega-3s (above the accepted minimum requirement) to slow aging and reduce disease requires further testing. In fact it is a common conundrum in the nutrition/metabolism research field as to what constitutes nutritional deficiency or excess, and whether compensatory nutritional alterations (supplementation or restriction of the ‘required’ amount, or simply removing an ‘excess’ or ‘deficiency’) affect healthspan or lifespan. Thus, by definition, causes and mediators of aging require not just the alleviation of a disease inducing state, but promotion of a health maintaining state that reduces disease risk, while increasing health span and longevity. To help address this question, a recent study investigated the effect of macronutrient balance on longevity in mice by testing 10 diet compositions at 3 energy densities, and found that mTOR signaling responded to the dietary protein content (increased mTOR signaling with more protein), raising the possibility that compounds like rapamycin which have significant longevity effects and alter amino acid signaling, may be modified by the background dietary protein and/or amino acid content. Alternatively, varied dietary compositions having different macronutrient proportions, individual nutrient restrictions, impaired nutrient metabolism, etc. could be modified to reduce signaling through pro-aging pathways, thereby increasing health and longevity. Whether this is the underlying mechanism(s) by which CR exerts its varied health benefits requires future study. However, manipulations of the diet by compound inclusion or individual nutrient restrictions, because they permit unrestricted intake, remains a type of intervention that more people will likely accept than CR as a change in their lifestyle.

## Autophagy and increased longevity and health

Aging and senescence are associated with accumulation of damaged proteins and organelles, mitochondrial dysfunction, and metabolic imbalance, especially in post-mitotic cells ^41– 48^. These pathologies further exacerbate cell injury, resulting in organ failure and decreased lifespan. While the oxidative stress theory of aging has been explored, except in limited examples using gene knockout or overexpression of antioxidant enzymes, there is a general lack of correlation between the levels of antioxidants and lifespan ^49– 55^. Since proteins and organelles are normally cleared by the autophagy-lysosomal activity, an emerging concept for regulation of aging and lifespan is that autophagy-lysosomal activity becomes deficient in aging ^32, 56– 67^.

In general, it has been shown that autophagy is regulated at many levels, by nutrient availability, oxidative and reductive stress, hypoxia and pathogenic molecules ^68, 69^. When this activity is unable to meet the demand for clearance of damaged intracellular macromolecules and organelles, pathologies emerge and progress. For example, perturbation of autophagy is evident in neurodegenerative diseases including Alzheimer’s and Parkinson’s diseases ^70, 71^. In yeast, worms, flies, and mammals, insufficient autophagy contributes to accumulation of protein aggregates and dysfunctional mitochondria, thereby leading to a decrease in lifespan and the pathogenesis and progression of various age-dependent chronic diseases ^56, 57, 59^. In addition, inhibition of autophagy leads to deterioration in mitochondrial function ^72, 73^; for example, ATG7-deficient skeletal muscles and pancreatic β cells accumulate dysmorphic mitochondria and exhibit defective oxidative phosphorylation ^73^. In addition PINK1 knockout mice exhibit mitochondrial dysfunction in striatum, liver, brain tissue, and primary cortical neurons ^74, 75^.

In a complementary series of studies, a role for the beneficial effects of stimulating autophagy in healthy aging has emerged based on data from longevity studies in various models ^31, 60– 62, 64, 65, 76, 77^. Rapamycin, which inhibits mTORC1 activity and activates autophagy, has been shown to extend lifespan in several model organisms ^76– 78^. CR and resveratrol have also been shown to extend lifespan through SIRT1-mediated activation of autophagy in worms ^31^, and genetic manipulations of autophagy also extend lifespan in animal models ^57, 60, 62, 64, 65^.

While autophagy is an attractive therapeutic target for increasing life and health span, key questions remain regarding our understanding of the fundamental mechanisms of autophagic regulation, as well as the impact of autophagy activation on lifespan and health span. Investigations of these key questions are essential to our ability to fully exploit this pathway for improving healthy aging, longevity, and associated drug development.

One critical aspect is already clear – that the fundamental mechanisms of autophagy regulation are context-dependent. For example, autophagic activation in response to oxidative stress is differentially regulated from autophagy activated by starvation. Determining the relevant common and specific autophagy mechanisms, and identifying the signaling pathways that modulate the level, location and selectivity of autophagy, are crucial for a better understanding of autophagy in aging. Regarding our understanding of the impact of autophagy on aging, age-related disease and longevity, we must take into account that current animal studies are predominantly undertaken using relatively pathogen- and nemesis-free environments. How we translate these studies to humans is still an unanswered question. Epidemiologic studies can give important clues in this regard, although pinpointing a specific contribution of autophagy on life and health span in human longevity studies remains a technical challenge.

An interesting and topical aspect is the extent to which individual variation in the autophagy pathway impacts healthy aging. Understanding the genetic variants in the human population which modulate autophagy is a largely unexplored aspect of genomic studies, yet will be essential as we enter the era of personalized health care.

Perhaps metabolomics, proteomics, or bioenergetics health indices need to be developed using imaging, accessible biopsies or body fluid to provide the much-needed paradigm shifts in our understanding of, and capacity for, monitoring human health conditions ^80– 82^. Additionally, autophagy activation may have differential impact on different organs in terms of their function and longevity. If improving health span is what we desire, then how to define health is not as simple as it appears. What one person defines as a healthy circumstance, another person may find unacceptable. Thus, we may have to be able to distinguish health and illnesses of different organs in any given person.

The emerging autophagy theory of aging holds tremendous promise, but is clearly a part of a bigger story, and integration into the existing paradigms should be considered at the outset. For example, autophagy can be included into the metabolism theory of aging, which includes metabolism of metabolites, reactive species, proteins, and organelles. Another example is the hormesis theory, which suggests that weak stress induces adaptive changes in the organism and may correlate with longevity ^30, 48, 55, 82, 83^, thus, as Nietzsche opined, what does not kill you may make you stronger.

## Aging-associated predictors of chronic disease

Expansion of adipose tissue during positive energy balance is a primary determinant of obesity and related pathophysiological sequalae ^84, 85^. In contrast to the long-held belief that pro-inflammatory signals exert a negative impact on metabolism, emerging studies suggest that proinflammatory signaling is required for adipose tissue remodeling and expansion ^85^. In fact, impairments in the pro-inflammatory response of adipose tissue were shown to increase ectopic lipid accumulation, glucose intolerance and systemic inflammation. In a related fashion, adipose tissue acquires a pro-inflammatory and senescent-like state with aging independent of adiposity ^86^. Although macrophage infiltration is thought to be responsible for much of the increase in inflammation with obesity, infiltration is lower with aging in the absence of obesity suggesting that pro-inflammatory responses to aging may be mediated to reduce ectopic lipid storage. Possible evidence for this, described in a recent review ^86^, indicates that the pro-inflammatory cascade with aging in adipose tissue is mediated through preadipocytes. It is possible that progenitor turnover, higher fatty acid levels, and toxic metabolites may contribute to fat cell senescence which then increases release of pro-inflammatory cytokines, increasing the potential for senescence to spread locally from cell to cell. It is important to determine whether the pro-inflammatory cascade that occurs with aging is derived from an aging-induced attempt to sequester lipids in relevant adipose storage sites as opposed to deleterious storage in ectopic sites such as skeletal muscle and liver.

As described in previous sections, CR extends lifespan in rodents and controversially in non-human primates as well ^29, 87^. At least part of the mechanism by which CR extends lifespan is through reductions in all-cause mortality. In fact, prolonged CR lowers body fat, slows rate of muscle loss, and lowers the incidence of neoplasia, diabetes, and cardiovascular disease in rhesus monkeys ^29^. In addition, reproductive endpoints and brain morphology are preserved with CR. Since CR has been initiated at an early age in most studies, future studies are warranted to understand the effects of CR when initiated in adulthood. It is particularly important to understand the effects of CR following prolonged positive energy balance in adult rodents and non-human primates. Since recent human studies have suggested that exercise training can ameliorate the negative consequences of weight gain, it would be particularly beneficial to observe the effects of exercise training (forced or voluntary) during periods of CR and weight gain. Observation of mitochondrial function during these periods of CR and caloric surplus combined with either exercise training or no training would also be of great value.

Dietary restriction of the essential amino acid methionine has been shown to increase lifespan in rodents ^38^ suggesting that restriction of total calories is not an absolute requirement for extending lifespan. While restricting fat and carbohydrate intake produces no extension of lifespan or improvements in markers of aging, improvements in the quality of carbohydrate consumption may be involved. Along these lines, a lower glycemic index (GI) diet can attenuate blood glucose excursions, lower postprandial lipemia, and decrease inflammatory markers. This is critical as many of these processes have been linked to age-related macular degeneration (AMD) and other aging associated diseases. Recent work by Uchiki *et al.* ^88^, using mouse and cell models, revealed that consumption of higher GI diets may increase risk for AMD and other aging diseases by increasing glycative stress-associated lesions in several different tissue compartments. The increase in advanced glycation end products (AGE) were due to a decrease in both the ubiquitin-proteasome system and lysosome/autophagy pathway, which are critical for degrading AGEs. Thus, it appears that one potential mechanism by which a higher GI diet can increase risk for AMD and other diseases is by altering cellular proteolytic activity. Future studies should be conducted to elucidate additional biological mechanisms by which consumption of a lower GI diet can promote health.

## Use and effects of mesenchymal stem cells (MSCs)

Significant changes in fat mass and distribution occur during aging and the redistribution of adipocytes in the elderly has been linked with a number of age-related disorders. Notably, about 15–50% of the cells in adipocyte tissue comprise preadipocytes that give rise to fat cells ^89^ and that play major roles in the mass and distribution of adipocyte cells during aging. Preadipocytes arise from adult stem cells in mammalian organs and one of the most important types of adult stem cell is the MSC. These are multipotent stromal cells that can differentiate into not only adipocytes, but also osteoblasts and chondrocytes as well as other cell types. Although MSCs have a high capacity for self-renewal, their ability to proliferate and differentiate decrease during aging, and with increasing passage number *in vitro* ^90, 91^. Considerable interest has focused on the mechanisms of MSC proliferation and differentiation that may provide novel approaches for tissue regeneration, a cornerstone of advances in interventional aging research.

Recent work by Vidal *et al.* ^91^ provides important insights into the senescence of MSCs that are central to organismal aging. To determine the potential use of MSCs in regenerative medicine, three different types of equine MSCs, bone marrow (BMSC), adipose tissue (ASC) and umbilical cord tissue (UCMSC), were evaluated for onset of cellular senescence in culture which has direct implications in their use for regenerative measures during aging, especially of fat and bone. It was discovered that BMSCs senesce after fewer population doublings than ASCs and UCMSCs demonstrating the more limited use of these types of MSCs for tissue regeneration. This also suggests that adipose and umbilical cord tissue may be preferable for tissue banking purposes although further studies on MSCs from different tissue sources are sorely needed in order to fully elucidate the potential for future therapeutic strategies in tissue repair during aging ^92^. Potential differences in methodology, however, could account for some of the reported results of limited capacity of BMSCs for tissue regeneration and additional studies will be required for optimization and standardization of cell culture conditions for the different types of MSCs. Also, yet unsolved is the differentiation potential of the various MSCs (BMSCs, ASCs and UCMSCs) at higher passage, since loss of differentiation potential could severely limit their ability to create new tissue for regenerative medicine and their application in the elderly.

A number of intriguing questions have arisen with respect to the mechanisms responsible for the attenuation of MSC proliferation and differentiation potential during aging. Among these remaining enigmas is whether senescence can occur at any stage of life ^86^, which is clearly important with respect to the role of MSCs during aging. It is also not yet clear if senescence can spread from cell to cell in fat tissue *in vivo* or whether cellular senescence is a key cause for metabolic dysfunction secondary to age- and obesity-related changes in adipose tissue ^86^. Future studies will also need to be directed toward determining if a senescence-like state can develop in terminally differentiated cells. Although there are suggestions that all of these venues are important with respect to MSCs, considerable additional research will be required before the role of MSCs in aging and their potential in tissue regeneration can be fully appreciated.

As aforementioned, MSCs are preadipocyte cells that give rise to new fat cells. The MSC progenitor cells can produce osteoblasts as well as macrophages that produce mesenchymal adipocyte-like default (MAD) cells important in age-related fat tissue redistribution and metabolic dysfunction. However, the variation in macrophage content during aging in subcutaneous fat tissue has shown little change while changes were noted between age and percent of macrophages in human omental adipose tissue ^92^. This suggests that additional studies are required and that MSCs and subsequent macrophage distributions and abundance during aging among fat deposits will require considerable future investigation.

Lastly, MSCs are also central to bone marrow fat during aging and in age-related diseases such as diabetes. It is noteworthy that fat occupies a significant proportion of bone marrow; although its role in aging and age-associated disease is not well understood. A recent investigation by Krings *et al.* ^93^ suggests that important changes occur in bone marrow fat not only during aging, but also in diabetes. MSCs give rise to different types of fat such as brown and white adipose tissue (BAT and WAT, respectively) and the metabolic phenotypes of bone marrow fat have characteristics of both types of adipose tissue. It was found that a decrease in BAT-like characteristics with aging and diabetes may contribute to age-related loss of bone remodeling and hematopoiesis that could have important implications in a number of changes that occur with aging secondary to the dynamics of MSCs in bone marrow ^93^. This may create avenues for therapeutic interventions for bone integrity during aging and in age-associated diseases in addition to regenerative medicine possibilities for skeletal tissue. However, future studies will be required to determine the role of MSCs in bone marrow during aging and metabolic diseases especially with respect to their lineage identity and differential adipose content in bone marrow.

Taken together, although a number of studies have eloquently revealed many of the roles of MSCs with respect to aging and age-related diseases, there are many avenues for additional studies to further reveal (1) the dynamics of cellular senescence *in vivo* and how this may limit potential for regenerative medicine approaches, (2) the behavior of MSCs from different tissue sources during aging, (3) the mechanisms for senescence of MSCs and how this impacts not only the aging process itself, but advances in intervention of aging and age-associated diseases, (4) the distribution and abundance of MSCs and macrophages during aging among fat depots, and (5) the role of MSCs in bone marrow and the importance of MSCs in contributing to maintenance of skeletal integrity in the elderly. Clearly many important advances have contributed significantly to our understanding of MSCs and their roles in aging; although many key questions remain that will provide fertile ground for future investigations.

## Telomeres relative to aging and energetics

Telomeres, the repetitive sequences at the end of chromosomes that buffer the genetic coding regions, suffer from attrition during successive cycles of DNA replication and cellular division. The telomerase enzyme, responsible for the maintenance of telomere length, is thought to be active in germ-line cells, but relatively inactive in differentiated tissues in humans. Thus, the gradual erosion of the chromosome ends is proposed to contribute to genetic instability and loss under conditions of replicative stress and over the course of life ^94^. When a critically short telomere length is reached, the cell enters replicative senescence and can no longer contribute to regenerative needs of the particular tissue ^95^.

Telomere length is variable between individuals at birth and with the proliferative demands of different body tissues, telomere length can vary between tissues within an individual ^94^. Previous research has shown that shortened telomeres are associated with a number of metabolic- and age-related pathologies (e.g. oxidative stress, inflammation) and diseases (cardiovascular, diabetes, cancer, obesity) ^96– 99^. Controlling for individual variations in telomere length by using intra-individual telomere length, estimated attrition is further associated with potential contributors insulin resistance, a proposed mediator of metabolic-related disease and aging ^100^.

From a broader comparative perspective, humans have relatively short telomeres yet age more slowly and live significantly longer than other mammals. Despite these interspecies differences, the observed associations between telomere length and attrition suggest there may be additional information to be learned by understanding the physiologic contributors to telomere attrition that overlap with aging modulation. In this regard, a recent review discussing the obesity-mortality association has highlighted a number of gaps in the current knowledge ^99^. For instance, there is a known association between excess body weight as measured by body mass index (BMI) and morbidity/mortality, yet there are exceptions where individuals with high-BMIs can have better than average health outcomes. Whether this BMI-mortality association is due to inter-individual differences in cellular aging rates, which may be captured through monitoring telomere change with body mass change, remains to be seen. Similarly, within individuals, whether telomere attrition is a composite outcome of age-sensitive biological mediators (oxidative stress, inflammation) that would reliably predict morbidity and mortality remains to be demonstrated. Along this same line, longitudinal studies of telomere dynamics within specific interventions to accelerate or retard aging (e.g., high fat diet feeding vs. dietary restriction), and including early life exposures and late-life health outcomes to better understand the “early life programming” effect in disease risk, could be informative. Such studies should include measures of caloric intake, energy balance, body composition, non-caloric nutritional factors associated with telomere length maintenance, stress exposure/protection, and co-morbidities in the outcomes. For the present, there is strong consensus that telomere shortening can contribute to cellular health and replicative capacity, and telomere length is correlated with a number of energetic- and age-associated negative health outcomes. Although it is not yet clear that telomere length or attrition is causative in these cases, particularly for mortality in humans, future research will require identifying the most relevant metric of telomere length (shortest single chromosome telomere length vs. population mean or overall distribution, etc.) and using such information to assess whether telomere attrition is more of a biomarker or causative factor in age-related phenotypes.

## Accretion and effects of body fat

Aging is generally associated with increases in total adiposity as well as waist circumference despite a lack of change or even a reduction in body weight (or concomitant decreases in lean mass) ^37, 101^. Positive energy balance, as resulted from subtle decreases in physical activity and basal metabolic rate that are not matched by decreases in energy intake, is suggested to be the cause for this increased adiposity ^101^. During advanced old age, while total amount of fat mass tends to decline or remains stable, fat is redistributed from subcutaneous to intra-abdominal visceral depots and to ectopic sites, including muscle (cardiac and skeletal), liver and bone marrow ^86, 89^. Fat redistribution and fat tissue dysregulaton with aging occurs across species and is associated with age-related diseases, lipotoxicity, changed metabolic variables and reduced longevity, with some of these changes more closely related to regional adipose tissue distribution than total fat mass ^89, 93^. These changes might be explained by the increased systemic free fatty acid exposure caused by impaired capacity of fat tissue to store lipid ^89^. Total or regional fat distribution with aging could have influence on morbidity and mortality risk, for example the removal of visceral fat significantly prolonged lifespan in rats ^101, 102^. In humans, weight gain and visceral adiposity is strongly associated with diabetes, atherosclerosis, thrombiosis, hypertension and other age-related diseases ^103^. It is also notable that there appears to be protective effects of aerobic fitness on all-cause mortality, even at high levels of adiposity. Poor aerobic fitness is a powerful predictor of all-cause mortality across a wide BMI range ^104, 105^.

Brown adipose tissue (BAT), which is related to thermal dysregulation and energy imbalance, decreases with aging in both animals and humans ^89^. However, it is unclear whether the decline in BAT is related to the white adipose tissue changes with aging. With aging, midfacial fat compartments have an inferior migration and volume shift ^106^.

Preadipocytes, comprising a significant proportion of cells in adipose tissue, are constantly giving rise to new fat cells throughout life, but with aging they are less capable of accumulating lipid than are cells from younger individuals, which may contribute to the increased abundance of small, insulin-resistant, dysfunctional fat cells ^89^. Preadipocytes also have decreased replication and adipogenesis, and increased proinflammatory cytokines and susceptibility to lipotoxicity with age ^86^.

Fat, which was once thought of as a largely biologically inert lipid storage depot, also plays an important role in the regulation of energy metabolism, thermoregulation, inflammation, and immune responses that is implemented through a number of adipokines derived from adipose tissue ^2, 93^. Adiponectin, a cytokine primarily originating from adipose tissue, has been shown to negatively correlate with many age- and obesity-related diseases and positively correlate with longevity in mice ^107, 108^. Similarly, leptin, which has been shown to modulate total body fat and visceral fat distribution, might play a causative role in the metabolic decline in aging independent of fat mass ^109, 110^. As in obesity, aging is frequently associated with increased fat tissue and circulating pro-inflammatory cytokines secreted by dysfunctional fat cells, including tumor necrosis factors-α (TNF-α) and interleukin (IL-6) ^86^, which in turn alter T lymphocyte subsets and attract mast cells and cause monocyte recruitment and macrophage activation ^89^. Macrophages infiltrate adipose tissue of obese animals and humans more extensively in visceral fat than subcutaneous fat. Animal studies show macrophages and pro-inflammatory factors increase with aging mainly in subcutaneous fat but remain constantly high in visceral fat throughout life, which implicates that subcutaneous fat gets dysfunctional with aging, potentially contributing to fat redistribution ^86, 89^.

Under CR, proportionally more body fat is lost than other tissue, which may contribute to alterations in systemic metabolic homeostasis, influencing factors at the interface of metabolism and inflammation ^37^. In aging studies utilizing non-human primates, CR lowers body weight, decreases fat mass and improves insulin sensitivity ^29, 37^. One of the most striking effects of CR in non-human primates is the impact on body composition, especially fat mass in the abdominal region ^29^. A study on rats has shown that CR improves carbohydrate metabolism in aging by decreasing visceral fat ^111^. Thus it is suggested that interventions that stimulate metabolism and/or activate WAT signaling hold great promise as mimics of CR ^37^. However, it is not clear yet whether CR is as effective in non-overweight individuals or certain stages of lifespan. Furthermore, it is advisable to titrate allocations in already fat-depleted animals in order to guard against drawing on lean tissue for energy since food intake steadily declines after middle age ^29^.

## The aging heart

Although other organs were discussed in the top 40 papers that we reviewed, the heart received particular emphasis. Congestive heart failure is an age-associated disease and is the direct result of defective cardiac aging ^112^. The results from these studies suggest that fibrosis is a common consequence of myocardial infarction (MI) in older adults, but less common in younger adults ^113^. The heart is a complex organ beating non-stop and is composed of 2 main cell types, cardiomyocytes and fibroblasts, along with pacemaker cells, Purkinje fibers, smooth muscle cells, resident macrophages, endothelial and epicardial cells, and a pool of resident stem cells. With aging, cardiomyocytes tend to either develop hypertrophy or wear and tear leading to necrosis. Fibroblast dysfunction with age leading to fibrosis, primarily contributes to diastolic heart failure in elderly individuals. Following a heart attack aging fibroblasts fail to develop stable scars and there is extensive left ventricle dilation and reduced survival in aging rodents and elderly individuals ^113, 114^. Novel strategies are required to reduce wear and tear in cardiomyocytes and preserve fibroblast function in order to form mature matrix in left ventricle remodeling in aging. Therapies are under investigation that stimulate cardiomyocyte regenerative capacity and limit fibrotic progress, but in the majority of cases, aging, the main confounding factor, is ignored.

Hypertension is highly associated with aging and congestive heart failure. Congestive heart failure significantly reduces cardiac output and limits functional capacity and quality of life. Age-related increases in the PR (PQ) interval (> 200 ms; distance on an ECG from the start of electrical conduction in the atria (beginning of p wave) to the start of electrical conduction in the ventricles (beginning of q wave) are positively associated with increased incidence of heart failure ^115^. The relationship between ECG P _wave_ to R _wave_ interval (PRI) and heart failure has important clinical implications suggesting that monitoring of PRI may be a cost-effective strategy to identify individuals at increased risk for adverse outcomes associated with aging. Additional large-scale studies are needed to more precisely identify the magnitude of PRI prolongation over the lifespan. Future human studies should incorporate younger participants to elucidate the relationship between PRI and heart failure. In addition, an important area of investigation will be to determine if lifestyle interventions such as exercise and/or energy restriction (in the presence or absence of weight loss) reduces age-associated prolongation of PRI and subsequent incidence of heart failure and atrial fibrillation. Further validation with echocardiographic data such as chamber size and left ventricular mass are also crucial for clinical utility of the PRI in predicting adverse outcomes in multiple race/ethnicities.

Control of inflammation is a widely studied approach for the treatment of MI, based on evidence from population-based studies. These studies suggest a direct role of inflammatory cytokines such as IL-6 and TNF-α on heart failure development ^116^. Over the last thirty years, use of anti-inflammatory treatments has been discouraged for the treatment of post-MI inflammation in heart failure pathology. The reason for this is that studies with older animals indicate that aberrant inflammatory responses – either defective or overactive –are associated with worse outcomes, in particular reduced collagen deposition in response to injury. Interestingly, the reverse of what is observed in young animals. Therefore, an anti-fibrotic or anti-inflammatory strategy needs to be used with caution in older individuals. For example, methyl prednisolone treatment has been reported to cause ventricular rupture in humans and specific cyclooxygenase 2 inhibitors in fact induce more MI events. TNF-α trials too have so far failed to meet the expectation of cardiologist for heart failure treatment ^117– 119^. Thus, in the last three decades anti-inflammatory approaches have not been shown to be effective. Therefore, novel targets with a major focus on the resolution of inflammation are necessary.

Lindsey *et al.* ^120^ showed that aging alters extracellular matrix remodeling and observed that middle aged (15 month-old) and older (23 month-old) CB6F1 mice had increased soluble protein fraction and decreased insoluble collagen compared to young mice. These structural changes are accompanied by increased end-diastolic dimension and ventricular wall thickness in aging mice with increased metalloproteinases (MMPs-3,8,9,12) and decreased inhibitory factors (TIMPs-3 and 4) expression. Thus, functional and proliferative capacites of fibroblasts are essential to maintain degraded matrix and to ensure that the structural integrity of the left ventricle is maintained in aging ^120^.

Age-related increases in fibrosis are also observed in C57Bl/6J sarcopenic and aging mice. Senescent (~32 month-old) C57BL/6J mice developed left ventricle structural changes characterized as interstitial fibrosis leading to increased left ventricular end diastolic dimension, decreased wall thickness, and decreased ejection fraction, indicating reduced contractile performance due to fibrosis.

Thus, collectively preventative strategies are required to prevent ventricular fibrosis in aging ^121^. While developing prevention strategies, the post-MI therapeutic treatments are needed to maintain survival of cardiomyocte and preserve fibroblast function mainly focusing on resolution of inflammation rather than inhibition in the reparative phase of remodeling of the aging heart.

## Mitochondria, reactive oxygen species, and cellular energetics

Denham Harman originally proposed the “free radical theory of aging” in 1956, which was later refined to the “mitochondrial theory of aging” in 1970’s. The common theme in both was that oxidative damage accumulates with age; inclusion of the latter implicated reactive oxygen species generated by mitochondria as the primary source of this stress. This theory has been intensively explored – some studies have provided results consistent with this theory, whereas the majority has not. For example, in some cases oxidative stress increased lifespan, and other reports have shown that increased mitochondrial DNA (mtDNA) mutation associated with decreased function, without a concomitant increase in oxidative stress, is sufficient for generating a premature aging phenotype. At the inception of both these ideas the central paradigm for the free radical field was “oxidative stress” in which the simple concept was that the aging was accelerated by an imbalance between oxidants and antioxidants. The hypothesis was later refined to encompass data that suggested that mitochondrial superoxide and hydrogen peroxide were uncontrolled “leaks” from electron transfer and mitochondria were the major source of reactive species in the cell. Since the 1970’s it is clear that both concepts are not supported by experimental evidence and cannot encompass the role of reactive species such as hydrogen peroxide, nitric oxide and electrophilic lipids as cell signaling molecules ^71, 122, 123^. Mitochondria are clearly not the major source of reactive species in the cell and whole families of proteins have now been discovered which show the controlled production of superoxide, nitric oxide, hydrogen peroxide and other reactive species with which the mitochondria interact in cell signaling cascades ^122, 124– 126^. Consequently, mitochondrial function may prove to have a more critical role in aging, not because of its generation of damaging ROS, but because of its role in cell signaling.

It is instructive to consider the evolution of mitochondria, which are ancient bacterial symbionts with their own genetic and protein synthesis systems, to understand the fundamental nature of mitochondrial signaling to the cell, sometimes called retrograde signaling ^127^. Each cell can contain hundreds to thousands of mitochondria each of which contains multiple copies of mtDNA. The mtDNA encodes genes essential for electron transport and oxidative phosphorylation. A unique aspect of mitochondria is that the proteins required for its multiple functions are encoded by both nuclear and mitochondrial genomes, requiring coordination and communication between the two cellular compartments. While reminiscent of its endosymbiotic origins, the mitochondrion retained many of the catalytic subunits required for electron transport and ATP synthase in its mtDNA. Hence, any mutation within the mtDNA has the potential to modify mitochondrial bioenergetics. In this respect, it has been proposed that prehistoric mutations in the mtDNA modulated the economics of mitochondrial production in the interrelated functions of ATP (energy), heat (thermoregulation), and superoxide (which can be converted to H _2_O _2_ and act as a signaling molecule) production, enabling our ancestors to successfully establish populations northward as they migrated from Africa. MtDNA mutations in northern migrants decreased caloric utilization for ATP generation while increasing that used for generating heat, creating a metabolic advantage for survival in colder climates. Although decreasing mitochondrial economy (in terms of ATP generation per calorie consumed), these changes were accommodated by changes in diet (increased caloric intake associated with animal fats). By comparison, mtDNA mutation that increased caloric utilization for ATP (thus decreasing energy lost to heat production) would be better adapted to low calorie diets and warmer climates (e.g., sub-Saharan Africa). In contemporary society, these prehistoric adaptations of mitochondrial economy can affect mitochondrial health and influence susceptibility to age-related disease in a setting of chronic positive energy balance ^72, 128^. In support of these concepts are molecular epidemiologic studies that correlate both human longevity and disease risk with certain mtDNA polymorphisms. More recently, it has been shown that mtDNA can directly influence susceptibility to heart failure and hepatic steatosis in mice, consistent with concepts of mitochondrial – nuclear genetic interactions ^129^. Hence, the role of the mitochondrion in aging is most likely multi-faceted due to its multiple cellular functions that include bioenergetics and signaling. More recently, studies have implicated mtDNA as a DAMP (damage associated molecular pattern) broadening its cellular functions to include immune response. The focus in the redox biology field has now shifted to understand why and how the “quality” of the mitochondrial population in cells declines with age and integrates concepts from autophagy, cell signaling and bioenergetics ^71, 130, 131^. Of particular interest is autophagy and mitochondrial biogenesis since they are both biological pathways that can link CR with healthy aging and bioenergetics ^37, 132^.

Hence, assessment of mitochondrial health in a dynamic fashion in our aging populations may provide a means for understanding the basis and progression of many forms of age-related diseases, including neurodegenerative diseases, diabetes, cardiovascular disease, liver disease, and cancer. This has now become possible with the development of measures of bioenergetic health from the leukocytes and platelets isolated from human subjects ^133, 134^.

## Conclusion

In conclusion, across forty of the most cited papers at the interface of aging and energetics published in the last four years, we were able to identify ten major themes of research suggestions. We have summarized them here for the interested investigator.

## Supplementary materials

Appendix A: Top ten most cited papers on aging and energetics in each of the four years that include 2010, 2011, 2012, and 2013.

The articles are organized in order of the number of citations within each year.

Click here to access the file.

http://dx.doi.org/10.5256/f1000research.5212.s35356
