# Supplementary material for: Aging and energetics’ ‘Top 40’ future research opportunities 2010-2013
Source: F1000Res. 2014 Sep 12;3:219. [Version 1] doi: 10.12688/f1000research.5212.1 (PMC4197746; doi:10.12688/f1000research.5212.1)
Supplement: Peer review history

# Peer review: Referee response for version 1

**DOI:** 10.5256/f1000research.5558.r6336

**Published:** 2014-10-07

**Authors:** George M. Martin

**Affiliations:** 1 Department of Pathology, University of Washington, Seattle, USA

In order to make conclusions about the current consensus on mechanisms involved in the very broad field of aging and energetics (broadly defined as the acquisition, storage and utilization of metabolizable energy by biological organisms), the authors have utilized a novel methodology for determining what researchers regard as the top 40 relevant publications over the period of 2011 through 2013, as ascertained via a broad search algorithm and ranked by the number of citations over that time period. Their analysis of these selected papers provide an interesting selection of some ten overlapping domains, including such ”old timers” as caloric restriction (now more accurately referred to as dietary restriction) and the new star of the field, mTOR signaling.

While this approach is an important guidepost to what has been discovered and, more importantly, as revealed by their analysis, what more needs to be done, the authors are encouraged to add an interesting and potentially informative control consisting of the *lower *40 most cited papers revealed by their search algorithm. Such an exercise has the potential to reveal highly novel ideas that, perhaps 10 or so years from now, could result in a new flowering of publications that might transform some segments of this exceedingly complicated family of nature-nature interactions.

I have read this submission. I believe that I have an appropriate level of expertise to confirm that it is of an acceptable scientific standard.

# Peer review: Referee response for version 1

**DOI:** 10.5256/f1000research.5558.r6110

**Published:** 2014-09-24

**Authors:** Derek M. Huffman

**Affiliations:** 1 Institute for Aging Research, Albert Einstein College of Medicine, Bronx, USA

Here, the authors summarize 40 of the most widely-cited papers on energetics and aging over the last 4 years by themes, including suggested priorities from those publications for future work. Overall, I think this article does a good job of highlighting and summarizing state-of-the-art aspects of several broad and complex topics. Discussion of the heterogeneity of the caloric restriction (CR) response was particularly warranted. The implementation and interpretation of CR studies requires careful consideration of among things, the intervention timing (early or later in life), diet quality, the control group ( *ad libitum* or mildly restricted) as well as the dose. Indeed, some strains that respond negatively to 40% CR, may respond differently at lower levels of restriction, in part due to reasons alluded to by the authors when discussing the potential risk of further fat depletion in already lean animals or humans. The authors also highlight some important recent observations regarding the role of specific nutrients (methionine) and botanical compounds (resveratrol) on aging, that do not involve calories *per se*. Of note, this has been further supported by a recent study that did not make the list ( Levine *et al*., 2014), linking protein intake with IGF-1 levels, cancer and mortality risk in younger, but not older humans.

Specific cellular mechanisms of interest are also discussed, with a particular emphasis on mTOR signaling in modulating organismal aging, and its interplay with nutrients and hormones. Rapamycin studies have driven much of the interest in this area, but metformin, which is not discussed here, is frequently and safely used clinically to treat diabetes, and may act in part via similar pathways, and has been linked to less cardiovascular disease, cognitive decline, cancers and improved survival in humans (see Bannister et al.,  2014). Overall, this is a well conceived and timely review of recent highly-cited papers linking aging and energetics that deserves revisiting in another 4 years.

I have read this submission. I believe that I have an appropriate level of expertise to confirm that it is of an acceptable scientific standard.

# Peer review: Referee response for version 1

**DOI:** 10.5256/f1000research.5558.r6106

**Published:** 2014-09-22

**Authors:** Holly M. Brown-Borg

**Affiliations:** 1 Department of Pharmacology, Physiology and Therapeutics, University of North Dakota, Grand Forks, USA

This is an interesting manuscript describing top research priorities in the aging and energetics field based on ideas extracted from recent papers. The title and abstract are appropriate and represent the information presented in the body of the manuscript. The basis of the paper is atypical in that the data generated are not assay-based but literature-based. The design is again, different but reasonable based on the outcomes to be generated. The data generated are ranked to identify the most highly cited papers in the past 4 years (2009<2014) and then further narrowed to 40 highly cited papers. The authors identified the top ten areas related to aging and energetics focusing on recommendations within these papers as to where further work should be focused. These areas included the CR longevity response, nutrient effects beyond energy, autophagy, mitochondria, ROS and cellular energetics and age-associated predictors of chronic disease among others. The recommendations for continued work in the areas covered were directed, insightful, and addressed strengths and weaknesses of the published studies and the area in general. Overall, the manuscript was well-written and provided important direction to those in the field in a relatively unbiased manner.

I have read this submission. I believe that I have an appropriate level of expertise to confirm that it is of an acceptable scientific standard.
